# Hemp-Derived CBD Used in Food and Food Supplements

**DOI:** 10.3390/molecules28248047

**Published:** 2023-12-12

**Authors:** Michaela Bartončíková, Barbora Lapčíková, Lubomír Lapčík, Tomáš Valenta

**Affiliations:** 1 Department of Foodstuff Technology, Faculty of Technology, Tomas Bata University in Zlin, Nám. T.G. Masaryka 5555, CZ-760 01 Zlin, Czech Republic; m_bartoncikova@utb.cz (M.B.); tvalenta@utb.cz (T.V.); 2 Department of Physical Chemistry, Faculty of Science, Palacky University Olomouc, 17. Listopadu 12, CZ-771 46 Olomouc, Czech Republic

**Keywords:** hemp, CBD, food products, cannabinoids, health benefits, functional properties, nutrition

## Abstract

*Cannabis sativa* L., a plant historically utilized for textile fibers, oil, and animal feed, is progressively being recognized as a potential food source. This review elucidates the nutritional and functional attributes of hemp and cannabidiol (CBD) within the context of food science. Hemp is characterized by the presence of approximately 545 secondary metabolites, among which around 144 are bioactive cannabinoids of primary importance. The study looks in detail at the nutritional components of cannabis and the potential health benefits of CBD, encompassing anti-inflammatory, anxiolytic, and antipsychotic effects. The review deals with the legislation and potential applications of hemp in the food industry and with the future directions of cannabis applications as well. The paper emphasizes the need for more scientific investigation to validate the safety and efficacy of hemp components in food products, as current research suggests that CBD may have great benefits for a wide range of consumers.

## 1. Introduction

*Cannabis sativa* L., an annual dioecious flowering plant, has been utilized for millennia as a raw material for textile fibers, oil, and animal feed. More recently, it has been recognized as a potential food source due to its rich content of medicinally important substances, particularly bioactive cannabinoids and alkaloids. These cannabinoids are terpenoid compounds that accumulate mainly in the cavities of cannabis trichomes [1,2,3,4].

However, the use of cannabis, particularly in the food industry, is a subject of much debate due to its psychoactive effects and the variability in the cannabinoid content of individual plants. The taxonomic division of this plant is complicated due to its genetic variability [1,5]. The genus *Cannabis* is divided into three main species: *Cannabis sativa*, *Cannabis indica*, and *Cannabis ruderalis* Janisch [6]. Based on the relative ease of crossing species, a monotypic classification has been made in which sown cannabis (*Cannabis sativa*) is divided into a total of five chemotypes distinguished by their cannabinoid profiles, i.e., the ratio of tetrahydrocannabinol (THC) to cannabidiol (CBD) content [1,5,6,7]. Chemotype I consists of the drug-type plants with a predominance of Δ9-THC. Chemotype II includes the plants with a high number of cannabinoids and with intermediate characteristics between drug-type and fiber plants. The fiber plants (chemotypes III, IV, and V) are characterized by the predominance of non-psychoactive cannabinoids and a great potential to produce sustainable fibers. The last chemotype, V, consists of fiber plants which contain almost no cannabinoids [8].

The primary metabolites present in cannabis mainly include amino acids, fatty acids, and steroids. The secondary metabolites consist of phytocannabinoids, flavonoids, terpenoids, lignans, and alkaloids. Phytocannabinoids are among the most extensively studied compounds in cannabis. The discovery and subsequent understanding of their biosynthetic pathway are crucial for identifying the individual compounds present in each plant [1,9].

Currently, a steady expansion of the range of cannabis products, including cannabis-infused foods, has been observed [10,11,12]. Food products that declare a cannabinoid content are made by adding hemp oil or hemp meal derived from the seeds and, where appropriate, cannabis extracts. However, the THC content must not exceed the permissible limits, prescribed by the legislation [11,13,14,15]. In Europe, the use of varieties of *Cannabis sativa* L. in the production of foodstuffs is permitted where the (-)-delta-9 trans-tetrahydrocannabinol (∆9-THC) and (-)-delta-9 trans-tetrahydrocannabinolic acid (∆9-THCA) content in the flowering parts or in the fruit tips from which the resin has not been removed does not exceed 0.2% of the dry matter [16].

This review aims to address a comprehensive overview of the primary and secondary metabolites present in the cannabis plant, with a particular emphasis on phytocannabinoids. The review will cover the biosynthetic pathway of cannabinoids, their synthesis and stability, functional and nutritional properties, as well as the effect of storage conditions. The current legislation governing the recreational and medical use of cannabis will also be discussed. In this context, we will focus on the rising consumer interest in cannabis-infused foods and the issues associated with using hemp in the food products. Finally, the review will explore the current state and future directions of potential cannabis applications in the food industry, discussing both the opportunities and challenges it presents. The objective of this review is to contribute to the ongoing debate about the practical usage of cannabis, particularly in the food industry, and provide a critical evaluation of the current state of knowledge in this area.

## 2. Cannabis Compounds: Chemical Composition, Synthesis, and Stability

### 2.1. Cannabis Compounds

The number of substances identified from the cannabis plant has been steadily increasing over the past few decades. In 1980, 423 cannabis compounds were known [9]. At present, this number has increased to 545 known compounds. The cannabinoids are the main constituents of the greatest importance.

#### 2.1.1. Cannabinoids

**Figure 1:**
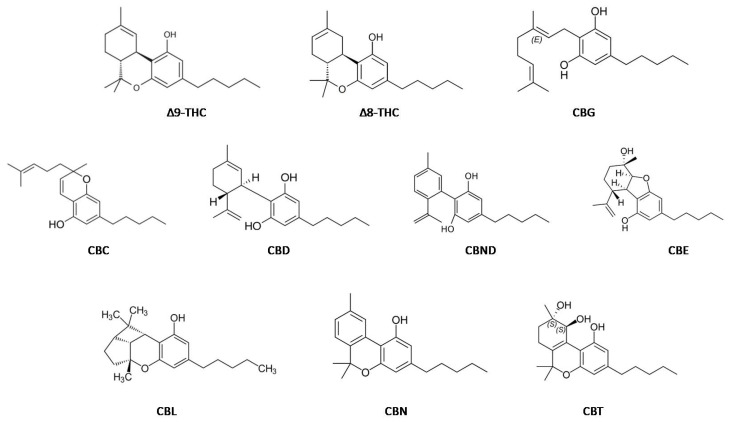
Cannabinoid compounds (adapted from Piscitelli and Di Marzo, 2021) [21].

The term cannabinoid refers not only to the chemicals isolated from cannabis plants, which contain a typical terpeno-phenolic C21 skeleton in their structure, but also to their possible plant-derived derivatives called phytocannabinoids. So far, a total of 104 phytocannabinoids have been isolated, which are divided into 11 types (Figure 1): (-)-delta-9 trans-tetrahydrocannabinol (Δ9-THC), (-)-delta-8 trans-tetrahydrocannabinol (Δ8-THC), cannabigerol (CBG), cannabichromene (CBC), cannabidiol (CBD), cannabinodiol (CBND), cannabielsoin (CBE), cannabicyclol (CBL), cannabinol (CBN), cannabitriol (CBT), and other cannabinoids [10]. The main representative of cannabinoids in the cannabis plant is Δ9-THC. It is a highly lipophilic substance, which, thanks to this property, easily penetrates the blood–brain barrier. In its pure form, it has strong hallucinogenic effects, but it has also been shown to be extractable from CBD, as the psychotropically inactive, crystalline component of the cannabis plant [17,18,19]. The naturally occurring form of THC is a mixture of two precannabinoids: ∆9-tetrahydrocannabinolic acid type A (∆9-THCA-A), which has antiproliferative, antispasmodic, neuroprotective, antioxidant, and anti-inflammatory effects, and type B (∆9-THCA-B). These two acids are positional isomers that differ in their physical properties, in the concentrations in which they can be found in different plant tissues, and their stability to decarboxylation. ∆9-THCA-A can be found mainly in amorphous form and its decarboxylation occurs at 90 °C. On the other hand, ∆9-THCA-B is found only in its crystalline form and is stable at 90 °C [20].

##### Cannabidiol (CBD)

Cannabidiol is the main cannabinoid that does not have psychotropic effects. It is one of the most important phytocannabinoids in fibrous cannabis and its carboxylated form was also the first isolated precannabinoid. CBD and THC, as the most abundant phytocannabinoids in the cannabis plant (*Cannabis sativa*), have different biological profiles despite their structural similarities, and although CBD can be electrophilically cyclized using acids to THC, this reaction does not occur in plants and both compounds are formed by oxidative cyclization of cannabigerol acid [20,22].

CBD is an allosteric inhibitor of the CB1 receptor, but it also acts through several different mechanisms. The first studies that looked at the biological activity of CBD were triggered by its modulatory activity on cytochromes and the possible potential for drug interactions [20,23,24].

Cannabidiol (CBD) has a wide range of effects on the human body due to its interaction with the endocannabinoid system, which is responsible for maintaining balance and homeostasis in the body. The following list presents some of the key effects of CBD on the human body:

1. Anti-inflammatory effects: CBD has been studied for its anti-inflammatory properties. It interacts with receptors of the endocannabinoid system, which may reduce inflammatory processes in the body and promote cell regeneration. This can be useful for individuals suffering from chronic inflammatory diseases such as arthritis or Crohn’s disease [25].
2. Analgesic effects: CBD can act as an analgesic, reducing pain perception and providing relief from various types of pain, including neuropathic and chronic pain. Studies show that CBD can affect receptor pathways involved in pain perception.
3. Anxiolytic effects: CBD has been studied for its potential to reduce anxiety and stress. It has a calming effect on the central nervous system and may help improve mood and reduce stressful states [26].
4. Antioxidants: CBD has strong antioxidant properties, which means it can help neutralize free radicals and protect cells from damage. This can have a positive effect on overall health and reduce the risk of oxidative stress.
5. Neuroprotective effects: Studies suggest that CBD may have neuroprotective effects and protect the nervous system from damage and neurodegeneration. It may have potential in the treatment of neurological diseases such as Alzheimer’s and Parkinson’s.
6. Anticonvulsant effects: CBD has been approved as a treatment relieving chronic pain of some forms of epilepsy, especially in children with difficult-to-treat forms of epileptic seizures. It may reduce the frequency and severity of sudden seizures [27,28,29,30].

It is important to note that the effects of CBD on the human body can be individual and depend on the dose, the method of administration, and the condition of the individual. Most of the studies confirming the above-mentioned effects are based on preclinical and clinical studies in animals and small groups of human subjects, and further research is needed to fully understand the full effects of CBD on the human body.

The world has seen a record increase in CBD sales in recent years due to its wide range of health-promoting effects [31,32].

Although most studies of cannabinoid structures in recent years have focused primarily on the Δ9-THC structure, CBD is now at the forefront of many studies [33].

##### CBD Production

CBD production requires specific procedures and technologies to ensure the high quality and purity of the product. The different steps of the production technology are mentioned below:

1. CBD extraction from the hemp

The first step in the CBD production process is the extraction of cannabidiol from hemp plants. There are several extraction methods, including supercritical CO_2_ extraction, solvent extraction, and oil extraction. Supercritical CO_2_ extraction is the most common and is considered to be the gentlest method to obtain pure and concentrated CBD [34].

2. Purification and isolation of CBD

After extraction, CBD needs to be further purified and isolated from other cannabinoids and impurities. This can be carried out by fractionation, distillation, or chromatography. The aim is to obtain a highly pure CBD with minimal tetrahydrocannabinol (THC) [35].

3. Formulation of CBD products

Once isolated, CBD is often formulated into various products such as oils, capsules, creams, food supplements, or beverages. Formulation is important to ensure a stable and safe product with accurate dosing [36].

4. Quality and standardization

CBD production requires adherence to strict standards and procedures to ensure consistent product quality and potency. This includes testing for CBD, THC, and other cannabinoid content, as well as testing for impurities such as pesticides, herbicides, and heavy metals [37].

##### Cannabidiolic Acid

Cannabidiol or CBDA is among the first cannabinoid acids discovered, having been isolated in 1955. Together with CBD, it forms the main component of the glandular trichomes on the cannabis plant, where it makes up to 15% and has proven antiproliferative effects. CBDa is the form of cannabinoid found in raw unprocessed cannabis plants, while CBD is obtained during the extraction and decarboxylation processes [23,38].

##### Cannabinol (CBN)

CBN is the first phytocannabinoid to be isolated in pure form from the cannabis plant. It was originally thought to be the active psychotropic component of cannabis, which has since been disproved. It is present in negligible amounts in the fresh plant, as its formation is dependent on the oxidation of THC and increases with the progressive degradation of THC during storage. However, cannabinol itself is very resistant to oxidative degradation and, due to this property, has been used for the identification of cannabis with narcotic effects. It has a weak affinity for CB1 and CB2 receptors [20,23].

##### Cannabigerol (CBG)

It is a non-psychotropic cannabinoid, which was also the first cannabinoid synthesized [20,23].

It has only a marginal affinity for cannabinoid receptors, and thus acts mainly through mechanisms that are independent of them. CBG is the precursor for all other cannabinoids, including THC and CBD. During the growth of cannabis, it is present in the early stages mostly as the main cannabinoid, but later it is gradually converted to THC, CBD, and other cannabinoids. It is a potent antagonist of the menthol receptor TRPM8, which is used in the treatment of prostate cancer [20,23,39,40].

##### Cannabichromene (CBC)

Cannabichromene is another compound that has no psychoactive effects in humans. It has been suggested that CBC is the second most abundant phytocannabinoid in the cannabis plant, but this has been heavily overestimated due to the difficulty of separating CBC and CBD. Its concentration in cannabis is therefore actually significantly lower than that of the other representatives. It is mainly found in dried hemp in relatively large quantities since its synthesis is dependent on the decarboxylation of CBCA, which is induced by heating. Although this compound is found in almost all varieties of cannabis, its properties are not yet fully understood. The isoprene residue it possesses is oxidatively bound to the resorcinol ring. In many varieties, the presence of CBC is associated with the presence of ∆9-THC.

If cannabichromene is subjected to a thorough purification, we obtain a substance that is racemic and shows no activity with the cannabinoid receptor CB1. It is among the simpler representatives of natural phytocannabinoids that can be synthesized and, given its thermal and phytochemical instability, it is surprising that it has been determined in historical cannabis samples. It occurs naturally in gum or oil form and can be found to have anti-inflammatory, antimicrobial, and mild analgesic activity. Cannabichromene is one of the main cannabinoids that are characterized by blue fluorescence under UV light [20,23,41,42,43].

#### 2.1.2. Biosynthesis of Cannabinoids

**Figure 2:**
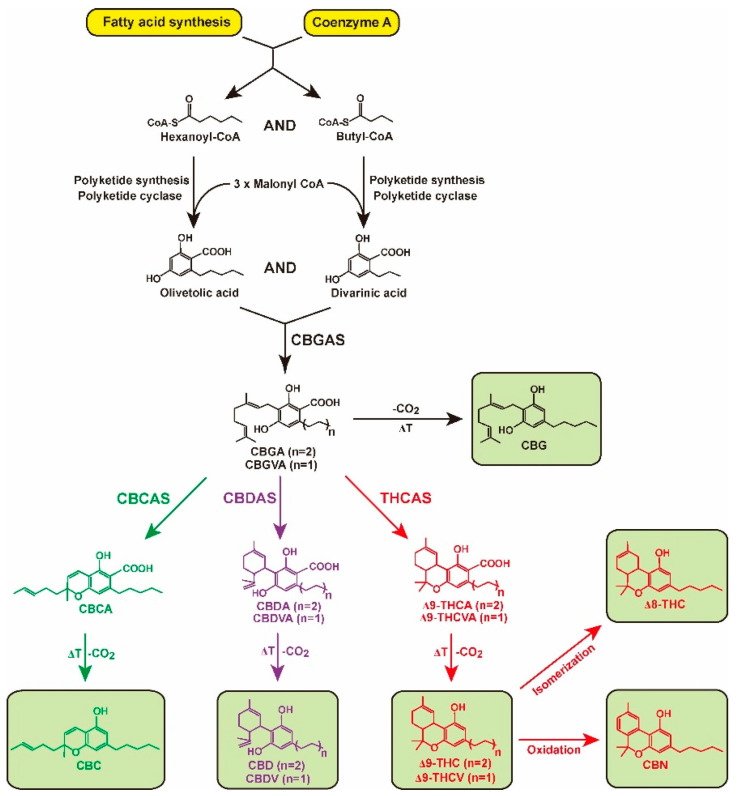
Biosynthesis pathway of cannabinoids (adapted from Kim et al., 2022) [48].

Thanks to several studies, we already know that the first step in cannabinoid biosynthesis is the formation of olivetolic acid, whose biosynthetic pathway has nonetheless not yet been fully elucidated. The biosynthesis of cannabigerollic acid (CBGA) occurs only in the presence of the precursors olivetolic acid and geranyl phosphate already mentioned. This reaction is catalyzed by the *prenylase* called *geranyl-diphosphate:olivetolate geranyltransferase* (GOT). This enzyme is responsible for catalyzing the first step in the synthesis of cannabinoids. At the end of this reaction, we obtain two compounds, CBGA and cannabigeric acid (Figure 2), which are precursors for the eventual synthesis of many other cannabinoids [40,42,44,45,46,47].

The subsequent transformation of CBGA, which is catalyzed by *tetrahydrocannabinol synthase*, leads to the formation of this acid (∆9-THCA). Due to the activity of *cannabidiol synthase*, the transformation of CBGA leads to the formation of cannabidiolic acid (CBDA), whereas the transformation of CBGA, which is catalyzed by *cannabichromenic acid synthase*, leads to the formation of this acid (CBCA). When these compounds are subjected to de-amylation, many products are formed, including ∆9-tetrahydrocannabinol (∆9-THC), cannabidiol (CBD), and cannabichromene (CBC). The same synthesis pathway can be observed for the remaining cannabinoid acids [41,45,46,49,50].

#### 2.1.3. Terpenes

Terpenes are a group of substances of which 120 have been identified in cannabis alone, of which 61 are monoterpenes, 52 sesquiterpenes, 2 are triterpenes, 1 is a diterpene, and 4 are terpene derivatives. They may be secondary or primary metabolites. If they occur as primary metabolites, they have the function of plant hormones such as cytosines or gibberellic acid and membrane stabilizers (sterols), whereas if they occur as secondary metabolites, they have a communication function and defense mechanisms [51].

In general, terpenes are responsible for the smell and aroma of cannabis. They are lipophilic compounds that readily cross membranes and are divided based on the number of repeating building units composed of five carbons called isoprene units. The content and distribution of each type of terpene in each plant depend on the conditions in which the plant is found or its maturity. The content of monoterpenes and sesquiterpenes has been identified mainly in the flowers, leaves, or roots of cannabis plants.

Of the monoterpenes, D-limonene, β-myrcene, α- and β-pinene, terpinolene, and linalool were the most prevalent. D-limonene, which is found in citrus, possesses anticancer and immunostimulatory properties; β-myrcene, which can be isolated from hop plants, is characterized by anti-inflammatory and analgesic properties. Sesquiterpenes and α-humulene are abundantly present in hemp extracts. Triterpenes are found in hemp roots, e.g., friedelin, or in hemp fibers, e.g., β-amyrin. These triterpene representatives are characterized by their antibacterial, antifungal, or anti-inflammatory properties [41,51].

Under standard conditions, a significant positive interaction between terpenes and cannabinoids was found, the main reason for which may be that the synthesis of mono- and sesquiterpenes takes place in identical glandular trichomes as the synthesis of cannabinoids. This synthesis may proceed in both directions for both compounds. The mevalonic acid pathway is characteristic for the synthesis of sesqui- and triterpenes, whereas the plastid-localized MEP pathway is required for the synthesis of mono-, di-, and tetraterpenes [50,52]. As reported by Brousseau et al. [53], visible LED light can stimulate biosynthesis in cannabis trichomes, enhancing tetrahydrocannabinol and terpene accumulation (but not CBD). By this photosynthetic way, the production of terpenes and other plant secondary metabolites can be optimized.

#### 2.1.4. Phenolic Compounds

Phenolic compounds belong to one of the most widespread groups of secondary metabolites. They include more than 10,000 different compounds such as phenolic acids, stilbenes, lignans, or flavonoids. The antioxidant properties of phenolic compounds can be expressed in plants and thus protect hemp from oxidative stress. In humans, the intake of phenolic compounds has been linked to a reduced incidence of chronic diseases such as cancer or cardiovascular disease.

Around 20 flavonoids have been identified in cannabis, mainly belonging to the flavone and flavonol subclasses. These include luteolin, kaempferol, quercetin, canflavin A, and canflavin B, which are methylated isoprenoid flavones unique to the cannabis plant.

A total of 19 stilbenes have been isolated from cannabis, including cannabistilbene I, IIa, IIb and dihydroresveratrol. Their main functional properties in plants are constitutive and inducible defense mechanisms, as well as growth inhibitors and sleep factors. They have anti-inflammatory and neuroprotective properties and are classified as antioxidants [34].

### 2.2. Synthesis and Stability of Cannabinoids

One of the common properties of cannabinoids with an acidic nature is that they undergo decarboxylation. Despite the fact that this is a fairly well-known piece of information, not enough weight is given to it, even though the active substance is the decomposition product of THCA. In some countries, regulations require the total THC equivalent to be reported as the sum of Δ9-THC and Δ9-THCA, defined by m(THC) + 0.877m(THCA). There is ample evidence to suggest that THC levels decrease over time [54,55].

The content of individual cannabinoids in samples can be used as a means of characterizing cannabis [56]. Examples include the ratio of CBN to THC, which is used in determining the age of a particular plant [57], or the ratio of CBDA to CBD, which can be used in assessing storage conditions [58].

**Figure 3:**
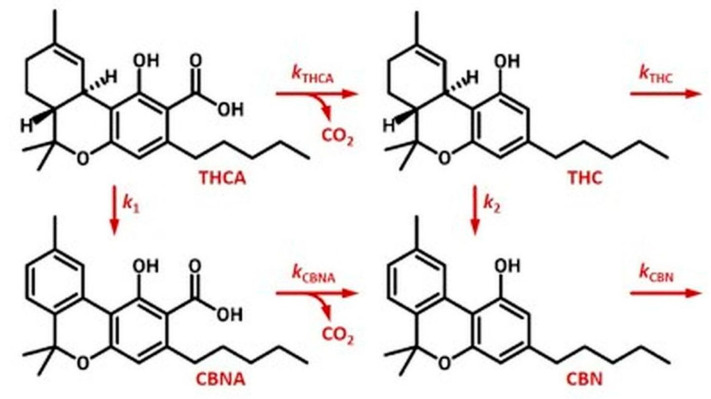
THC decarboxylation and degradation pathways (adapted from McPartland and Russo, 2001) [60].

As the dried form of cannabis is one of the most widely used variants in the market, it should be given due consideration, as it is here that a large proportion of the cannabinoids are present in the form of aromatic carboxylic acids, which are capable of undergoing decarboxylation even in the dried material [59]. An example of this process is the conversion of THCA to THC over time, where this change can be modeled by a pseudo-first order reaction system. In this process, A represents the cannabinoid in the carboxylated form, and N is the designation for its decarboxylated analogue. The specific example is tetrahydrocannabinolic acid (A=THCA), which changes to tetrahydrocannabinol (N=THC), as illustrated in Figure 3. In this way, the formation of cannabinol (CBN) and cannabinolic acid (CBNA) can also be reached [60].

In addition to the examples mentioned above, attention should also be paid to the conversion of CBD to THC in the presence of acids when exposed only to high temperatures [61]. Although very slow, decarboxylation occurs even at room temperature, and this is also related to the influence of storage conditions that may affect the composition of the cannabinoids present in the plant before extraction [62]. Decarboxylation has become one of the very important steps in the cannabis industry. In the recreational use of cannabis, decarboxylation occurs during smoking, vaping, or baking [63], but as the market for cannabis products is growing rapidly, there is a development of many new products that are based on extracts. Decarboxylation can be performed on the plant material prior to extraction or directly on the extracted oleoresin. To a large extent, the first method is preferred, as it offers two main advantages when combined with extraction. The first is the removal of the moisture present in the plant during decarboxylation and the second advantage is that cannabinoid acids are more polar in nature than their neutral analogues, hence their limited solubility in supercritical CO_2_ [64].

The decarboxylation process itself was reported as early as 1970 when Kimura and Okamoto [61] used elevated temperature (110 °C) to treat individual fractions of the cannabis plant to determine their concentration and distribution of THCA as a function of THC. Kanter et al. [60] described the decarboxylation process as one of the most important steps in the determination of THC content by HPLC. They determined the optimum conditions at 200 °C for a short reaction time (3 min).

At lower temperatures (80 °C), the conversion of THCA, CBDA, and CBGA to their respective neutral forms occurs at a relatively slow rate but increases with increasing temperature. When the sample is exposed to 120 °C for 90 min, THCA is completely decarboxylated, whereas at 160 °C the time required for complete decarboxylation is only 20 min. Thus, gradual decarboxylation leads to an increase in THC concentration. However, as the temperature increases, especially above 100 °C, THC reaches a maximum and the concentration starts to decrease and an increase in the concentration of CBN, the oxidation product of THC, is observed, which does not correspond to the amount of THC “lost”. This phenomenon has also been observed for CBD and CBG, where in both cases the sum of their molar concentrations in acidic and neutral form decreased by up to 90% over a period of 6 min at 160 °C. These findings suggest that the formation of unidentified by-products is possible together with the simultaneous evaporation of the neutral forms at higher temperatures. The boiling point of THC was determined to be 157 °C, and the boiling point of CBD ranges from 160 to 180 °C [5,65].

## 3. Cannabis Compounds: Functional and Nutritional Properties

Cannabis has been an important food source for mankind for thousands of years. The earliest evidence of its use comes from the discovery of hemp seeds in Chinese graves dating back to the third millennium. Hemp seeds are still commonly found in China today, where they can be bought on the street as a form of snack. Even though many parts of the cannabis plant are edible, the hemp seed is the most commonly consumed raw material. Due to the rediscovery of hemp’s nutritional benefits in recent years, cannabis products regained popularity in many countries around the world [66].

The hemp plant contains high amounts of protein, unsaturated fatty acids, and fiber. In cannabis, we encounter products of primary metabolism such as amino acids, proteins, fatty acids, and steroids [23]. Specifically, in the hemp seed we find all the essential amino acids that are important for maintaining a healthy lifestyle and proteins that are easily digestible and usable by the human body [19]. The main proteins found in the seeds include globulin, edestin, and albumin. Edestin accounts for about 60–80% of the total protein content. It is high in arginine and glutamic acid, relatively easy to digest, and rich in essential amino acids. Cannabidiol (CBD) is a non-psychoactive derivative of *Cannabis sativa* L. and one of the main cannabinoid substances.

Hemp seeds have an energy value of 500–600 kcal/100 g of the product, at roughly one quarter protein, one quarter carbohydrate, and one third fat. This composition varies from genotype to genotype and there are significant differences between them. Hemp seed is generally quite rich in polyunsaturated fatty acids. According to a study of seven different varieties of hemp (Bialobrzeskie, Felina 32, Tygra 75, Futura 27, Santhica, Fedora 17, Finola), the Finola variety had the highest linolenic acid content and the lowest content of oleic acid and saturated fatty acids: palmitic and stearic acids [67].

Cannabis proteins are generally a relatively valuable source of the sulphur-containing amino acids methionine and cysteine. They are also an excellent source of arginine, an essential amino acid with a positive effect on the human cardiovascular system. A study reports that just 50 mg of hemp seed covers 50–100% of the recommended daily allowance of several minerals, including copper, magnesium, and zinc, and provides up to 100% of the recommended daily allowance of vitamins A, D, and E [68].

Hemp seeds are also a suitable raw material to produce flour with quite valuable nutritional properties. Andrews et al. [69] reported that the flour from the Fedora variety contained moisture, protein, lipids, carbohydrates, and ash in the proportions of 7.9 ± 0.9%, 30.7 ± 1.2%, 13.6 ± 1.9%, 41.6 ± 2.5%, and 6.2–0.5%, respectively, while the content of total polyphenols quantified by the Folin–Ciocalteu method was relatively high. In laboratory experiments, it was found that up to a 10% addition of hemp flour to wheat flour did not affect the stability and strength of the dough but, on the contrary, significantly improved the nutritional value of the final product by increasing the protein and mineral content. In another published study [70], the addition of hemp flour to starch was used in the production of gluten-free bread. The result was an improvement in taste and color together with an increase in fiber and protein content, thereby improving the overall nutritional value of the product.

A practical problem in the processing of hemp seeds represents the fact that a very low but detectable amount of THC is present on the seed surface, which seems to be a contaminant from glandular trichomes at harvest time. For that reason, the hemp cultivating and handling processes should be performed in such a way to prevent the contamination with high levels of THC, ensuring the nutritional quality of the raw material [71].

In the food industry, hemp seeds are pressed mainly to extract oil and are also used to produce hemp flour. They are ground and serve as a quality source of vegetable proteins and fiber. Hemp seed flour has a superior nutritional quality compared to wheat flour, making it a suitable candidate for fortifying food products. It has a higher content of protein, fat, minerals, fiber, essential amino acids, and essential fatty acids compared to wheat flour [72]. Ground hemp seeds or hemp flour find application in the production of various food items. These include energy bars, where the high protein and fiber content can provide sustained energy release, and also flavoured yogurt, offering a unique taste and added nutritional benefits. In baked products, hemp flour can replace wheat flour, resulting in baked goods with a better nutritional profile [73,74,75].

Hemp oil produced from hemp seeds is also often used. Hemp seed oil contains a large amount of unsaturated fatty acids with proven cardioprotective effects, such as linoleic acid (18:2 omega-6) and α-linolenic acid (18:3 omega-3), which are represented in an optimal ratio of 2/3:1. This ratio explains the protective potential of hemp oil against cardiovascular disease, osteoporosis, and eczema. Compared to soybean oil, hemp seed oil is nutritionally more valuable: human daily nutritional needs for fats can be reached by the intake of three tablespoons of hemp oil [66]. The high level of polyunsaturated fatty acids in hemp seed oil indicates its usage as nutritious oil suitable for salad dressings; however, hemp oil is unsuitable for deep frying due to the high level of unsaturation [76].

Edible cannabis products can be used in many specific applications. Steinbach designed a production process for the manufacture of pralines and chocolates containing hemp seeds [77]. In 2009, hemp oil and hemp seeds were used by Shim to produce bread and confectionery products. Guang and Wenwei jointly developed a patent for the use of hemp flour in the production of functional foods, as its consumption increased high-density lipoprotein levels and balanced the levels of other glycerides [78,79,80]. Again, they produced hemp milk that did not turn bitter or discolored after the pasteurization process. A very important point in the use of cannabinoids in the food industry is to make the application of cannabis extracts to food as simple as possible [81].

While the nutritional and functional attributes of hemp components have been highlighted, it is crucial to consider the potential limitations and challenges associated with their use in the food industry. For instance, the variability in the concentration of bioactive compounds in different hemp varieties may affect the consistency and quality of hemp-based food products. Moreover, the extraction and processing methods used can also influence the nutritional value and functional properties of these products. Comparatively, while some studies have reported the health benefits of CBD, others have raised concerns about its safety and potential side effects. Therefore, it is essential to approach these findings with caution and consider them within the broader context of the existing research.

### Cannabinoid Intake in the Human Body

The ever-increasing interest in cannabis itself, but also in its compounds, has led to growing concerns about the safety of dietary supplements, dried cannabis, and foods containing cannabinoids. Over the past decades, many studies on the key properties of cannabinoids have opened many new possibilities for their use. In this context, understanding the effects of cannabinoids on the human body is very important.

Cannabinoids can enter the human body either through the respiratory tract or the digestive tract. The most popular products among consumers are mainly dietary supplements together with foods and beverages containing extracts or parts of *Cannabis sativa* L. [82]. When CBD and other cannabinoids are ingested, they are degraded in the acidic environment of the stomach and intestines by enzymes [83,84].

Oral administration of foods or dietary supplements containing cannabinoids results in an intense increase in hepatic metabolism. Application of cannabis products to the mucosa allows higher plasma concentrations to be achieved than with oral administration, although most drugs are administered in this way in medicine [84,85].

The metabolization of cannabinoids occurs in the liver and their complete removal from the body takes several days. In general, it is very difficult to estimate the time it takes for their breakdown to occur, as it is mainly influenced by the balance between plasma and adipose tissue in a particular individual. Ingested CBD is not metabolized and is excreted in the feces [16,82,84,86]. According to the currently available literature, there is no evidence to support that excessive use of cannabinoids leads to overdose or eventual death, but there are reports that excessive use can cause coma in children [16,87].

There are still many unknowns and concerns about the effects and potential interactions of cannabinoids in the human body. In order to ensure food safety, it is important to understand the pathways of transformation and degradation of cannabinoids in the context of the consumption of products containing them, especially in terms of toxicity. Many more studies are needed to address the usefulness of these products and their possible adverse health effects with long-term use, which are still relatively unclear, especially for cannabinoids, which occur in lower concentrations than CBD and are therefore less well known [10,11,12].

## 4. Cannabis Legislation

In many countries, the words “hemp” and “cannabis” are associated with “illegal drugs”, and hemp products stay in a “grey zone”, of which the legality is not clearly defined. However, the regulations of the products considerably vary depending on the country.

In the USA, the 2018 Farm Bill removed industrial hemp with THC < 0.3% from the definition of marijuana, and in the same year (2018), the Food and Drug Administration (FDA) approved the first medicine containing CBD for the treatment of seizures associated with two rare and severe forms of epilepsy, Lennox–Gastaut syndrome and Dravet syndrome. Some cannabis food products (e.g., tea, coffee, and chocolate) are already on the markets of some USA states after the states deregulated CBD, although it is still not approved at the federal level. In Canada, cannabis is nationally legalized, but phytocannabinoids, including CBD, are on the Prescription Drug List and cannot be legally sold in self-care natural health products and cosmetics. In China, there has been a long cultivation tradition of hemp; however, recent national regulations forbid any use of CBD in food and cosmetic products. These Chinese regulations significantly limit the development of the hemp industry on a large scale [51].

Within the Asian market, the landscape governing CBD-infused foods and dietary supplements is circumscribed by specific regulatory frameworks. Regulatory authorities maintain exhaustive positive or negative lists delineating permissible substances for inclusion in food products or for non-pharmaceutical applications. In the Japanese jurisdiction, cannabis-treated seeds find inclusion on the “non-drug list“. Notably, CBD oil, derived from the mature stem and seed of the cannabis plant, assumes a non-cannabis classification. This categorical distinction facilitates the importation of CBD products, specifically THC-free CBD oil, in alignment with guidelines promulgated by the Japanese Ministry of Health, Labour, and Welfare in 1999. Furthermore, the importation of chemically synthesized CBD is admissible, contingent upon the importer’s capacity to substantiate its non-classification as cannabis.

In South Korea, the inclusion of hemp seeds as a food ingredient is permissible, contingent upon the complete removal of shells, encompassing bracts and outer epidermis. Venturing into Southeast Asia, Thailand has recently instituted regulations designed to foster the cultivation of hemp as an economically viable crop. This regulatory framework expressly authorizes the incorporation of CBD oil, exclusively sourced from hemp seeds, in foods and dietary supplements. It delineates explicit conditions governing the utilization of hemp or *Cannabis sativa* (*C. sativa*) seeds, hemp seed oil, and hemp seed protein across diverse food categories, inclusive of dietary supplements. Additionally, the regulations establish stringent maximum limits for THC and CBD content, thereby ensuring the safety and regulatory compliance of these products [88].

In China, a historical hemp cultivation tradition prevails. The Chinese regulatory authority officially acknowledged cannabis as a botanical constituent suitable for both alimentary and medicinal applications in the inaugural iteration of the “List of Botanical Ingredients Recognized for Food and Medicinal Use” released in 2002. However, antecedent to future limitations across various product categories, four cannabis-related raw materials, including CBD, were preemptively included in the inventories of proscribed ingredients and raw materials for cosmetic formulations. Presently, national regulations in China prohibit the inclusion of CBD in both food and cosmetic products, imposing substantial constraints on the expansive development of the hemp industry [88].

Within Latin America, regulatory frameworks addressing the industrial and therapeutic applications of hemp or cannabis exist. Notably, Uruguay and Ecuador have enacted distinct regulations pertaining to the utilization of hemp seed protein and CBD in food products. In Uruguay, Decree No. 19/2020 amended the National Bromatological Regulation, permitting the incorporation of hemp seed protein derived from industrial hemp variety C. sativa in food items. The regulation stipulates a maximum THC concentration of 10 mg/kg for the powdered ingredient obtained through the processing of residual products from oil extraction. Conversely, Ecuador permits the use of all non-psychoactive cannabis parts or their derivatives in processed foods and food supplements, provided the THC concentration in the final product remains below 0.3%. Non-psychoactive cannabis or hemp, encompassing oils, resins, tinctures, crude extracts, or other technological innovations derived from non-psychoactive plants with a THC content below 1%, is defined as raw materials for the production of finished products [88].

In Australia and New Zealand, ministerial approval in 2017 resulted in an amendment to the Australia New Zealand Food Standards Code, enabling the commercialization of low-THC hemp seeds (*C. sativa*) for human consumption as food items or ingredients. The stipulation mandates that the only cannabinoids present in or on the seeds must occur naturally. Subsequent to this modification, amendments to various food and drug regulations were endorsed, taking effect in November 2018. These revised regulations delineate explicit conditions for the usage of hemp seeds, encompassing extracts, oils, and beverages, with prescribed maximum limits for THC and CBD content [88].

In the European Union (EU), the seeds of cannabis plants may be used for food purposes; the other parts of the plant and their extracts are designated as “novel foods” in accordance with EU Regulation 2015/2283 of the European Parliament and of the Council of 25 November 2015. This regulation defines the term as a food that has not been traditionally consumed in the EU before 15 May 1997. This group includes foods with a novel or modified molecular structure, foods that have been derived from products produced by microorganisms, fungi, or seaweed, and substances extracted from them. Hence, extracts of seeds of *Cannabis sativa* L. var. *sativa* and products thereof containing cannabinoids are considered “novel foods” [89]. In 2019, CBD was added to the category of “novel foods” and subjected to the authorization by food safety authorities which must be obtained before the launch to the EU market. Nonetheless, after the manufacturers had applied for this authorization, the process was temporarily suspended and some EU states began to withdraw the CBD products from the market, although others still allowed their sale. At present, CBD falls under different regulations depending on the nature of the products and their composition [90].

In Czechia (EU), CBD products are legal as cannabidiol is not classified as a narcotic or psychoactive substance. A clear indicator of legality is therefore primarily the THC content. The basic distinction between technical and “regular” cannabis is regulated by Government Regulation 463/2013 Coll. on the lists of addictive substances. According to it, a registered variety in which the psychoactive THC content does not exceed 1% of the dry weight of the plant can be considered technical cannabis. If the dry weight of cannabis exceeds this 1% limit, it is an addictive substance, the possession, use, or sale of which is illegal. According to Act 167/1998 Coll. on the lists of addictive substances, technical cannabis can be freely purchased, stored, and further processed [91].

In Switzerland, national legislation permits the inclusion of CBD in alimentary items and dietary supplements. Food products encompassing cannabinoids, such as CBD, extracts of *Cannabis sativa* (*C. sativa*), and derivatives with cannabinoids (e.g., hemp seed oil with added CBD, dietary supplements with CBD), fall under the classification of novel foods. Consequently, they necessitate licensing from Swiss authorities or approval from the European Commission prior to market introduction. Conversely, products derived from C. sativa or plant parts established as safe and significantly consumed in the EU before 15 May 1997 are exempt from novel food categorization in Switzerland. This exemption notably applies to hemp seeds, hemp seed oil, hemp seed flour, and defatted hemp seeds. Additionally, herbal tea derived from cannabis plant leaves in Switzerland is not considered a novel food item. Its use for flavoring food products is exempt from licensing requirements, provided it is employed as an aqueous infusion and not in concentrated or syrup form. Swiss legislation further prescribes explicit labeling regulations for CBD and establishes permissible THC levels in food products [88].

In the UK, regulations governing the marketing of CBD-infused foods and dietary supplements are applicable in England, Wales, and Scotland. Oversight of these regulations is administered by the Food Standards Agency (FSA) for England and Wales and Food Standards Scotland (FSS) for Scotland. The FSA classifies CBD extracts under the UK novel food regulation, necessitating novel food applications for product approval from the CBD industry. In contrast to the EU, the FSA allows businesses to continue selling CBD products that were already on the market as of 13 February 2020, with the stipulation that these products remain safe, correctly labeled, free from THC or any other substance regulated under drug legislation, and associated with a novel foods application submitted before 31 March 2021, which was subsequently validated. Conversely, the FSS prohibits the sale of ingredients classified as novel food in the Scottish market until full authorization is granted. However, applications should be submitted to the FSA without the requirement for a separate application to the FSS [88].

The current regulatory landscape of CBD legislation poses a multifaceted challenge necessitating critical examination by pertinent reference sources. The divergence in regulations and criteria concerning acceptable plant parts for food ingredients, as well as maximum THC limits, introduces considerable uncertainty. The situation is further compounded by distinct interpretations and implementations of rules by national authorities regarding isolate, synthetic, and full-spectrum CBD. In this evolving regulatory milieu, it is imperative to address these challenges and strive for lucid and consistent interpretations of prevailing legislation, a task pertinent not only to researchers but also to companies necessitating informed adaptation of marketing strategies. Nevertheless, the absence of clear and consistent regulations may constitute a barrier to unequivocally presenting legislative aspects of cannabis products and their integration into the food industry.

On a global scale, prevailing regulations underscore the escalating acknowledgment of the nutritional and functional attributes of hemp. However, they simultaneously emphasize the necessity for regulatory oversight to safeguard the safety and quality of cannabis products. A judicious legislative policy can facilitate the cultivation and utilization of the cannabis plant across diverse sectors, including the food industry, medicine, recreation, and other domains of human activity. Nonetheless, the absence of robust regulations may engender the illicit misuse of psychoactive cannabinoids and their derivatives [88].

## 5. Conditions of Cannabis Compound Use and Storage

In recent years, there has been a growing interest in hemp seeds and products containing hemp. With the growing popularity of these products, the range of products in the retail network is naturally expanding, with the most popular products being those made from hemp flour or containing hemp seeds or oils. The benefits of using these raw materials include their nutritional value and potential medicinal properties. However, the amount of these raw materials that can be used is determined by the cannabinoid content of the final product, and the ∆9-THC content must not exceed the permitted limits [13,14,92,93].

Currently, there is competition between food manufacturers to produce the greatest number of cannabis products. Many variables affect the possibilities and limitations of the use of cannabinoids in the food industry. It is necessary to regulate the limits not only of THC but also of other cannabinoids in the food products and to characterize precisely the properties of single cannabinoids. The effects of these compounds, as known to date, suggest that they could serve as alternatives to some conventional medicines. This is considered a significant potential benefit. However, obstacles to the development of this type of food are misinformation and an unjustified amount of prejudice. But given a number of reports on the positive effects of cannabinoids, the fear of adding them to the food seems to be unfounded [10].

Cannabis in the food industry is a very difficult matter due to the variable cannabinoid contents in different plants. The concentration of individual cannabinoids in hemp oil depends mainly on the variety and the way the seeds are cleaned [45]. According to a study by Hazekamp and Fischedick [94], the concentration of cannabinoids in plants of the same species grown in different parts of the world has been shown to vary by more than 25%. This variability is a significant limitation as it can affect the consistency and safety of food products. To mitigate these differences, it may be possible to consider controlling varieties and cultivation methods to ensure greater homogeneity or to mix the extracts from specific plants in such a proportion as to obtain the necessary homogeneity.

Regulations governing the permitted levels of cannabinoids in food vary from country to country (as discussed in Section 4). In most cases, the restrictions mainly concern ∆9-THC but do not include ∆9-THCA, which is converted to ∆9-THC after heat treatment. The legal limits are usually given in mg/kg (ppm) of the final product [10]. In Europe, varieties of *Cannabis sativa* L. with a total content of ∆9-THC and ∆9-THCA in the flowering or fruiting parts of the plant from which the resin has not been removed not exceeding 0.2% of dry matter may be used for food production purposes [16]. The use of other parts of the plant can be dangerous as the cannabinoid content varies across the plant concerning variety and growing conditions [89].

### 5.1. Use of Cannabis Extracts and Emulsions

Hemp extracts are characterized by their resinous, oily structure and easy solubility in organic solvents, fats, and alcohols. The choice of the form in which the cannabinoids can be added to the final product is very important so that they dissolve easily and do not affect the final product. Extracts containing CBD are usually readily dissolved in edible oils such as coconut or olive oil; CBD absorption in the digestive tract is enhanced by its incorporation in high-fat food. Conversely, CBD oil–water emulsions are used in the production of tinctures, soft capsules, or beverages. The use of this type of emulsion requires the use of surfactants—emulsifiers which may include polysaccharides, proteins, or phospholipids—where the choice of a particular emulsifier depends on the type of emulsion, the composition of the oil, and, finally, the ionic strength of the aqueous extract. The production of solid products is quite difficult due to the oily nature of hemp extracts. This problem is solved by using excipients that form a lipid matrix, which then allows the controlled release of cannabinoids and prevents their degradation [95,96,97,98].

### 5.2. Storage Conditions of Cannabis Products

Given the lipid nature of cannabinoids, they are quite susceptible to oxidation, which poses a risk mainly to their storage stability. Another equally important issue for manufacturers is to maintain a homogeneous concentration in each batch of the product. Regular control of the water activity in the products and proper packaging are non-negotiable measures that improve the quality and shelf life of the products [12].

Knowledge of the changes that occur when hemp oil is stored under different conditions is very important when considering its possible application in the food industry. As noted by Rupasinghe et al. [73], hemp oil is characterized by its susceptibility to rancidity, which can be caused by heat and storage for a long time. Acid cannabinoids, such as cannabidiol acid, are found in the composition of hemp oil and are present in relatively high concentrations in the oil. If we want to assess storage conditions, it is a good idea to pay attention to the ratio between CBDA and CBD. Any long storage time and incorrect storage conditions may result in the degradation of CBDA to CBD and thus change their ratio [65,99].

When raw materials are exposed to high temperatures, e.g., during drying or burning, the cannabinoid content changes. As a result of these processes, non-psychoactive carboxylic acids are converted to neutral cannabinoids by decarboxylation [99,100,101]. ∆9-THC, which is derived from ∆9-THCA, is converted to CBN as a result of decarboxylation due to the action of light and oxygen in oxidation processes [95]. The decarboxylation of these acids takes place only under the influence of temperature, and no enzymes are involved, i.e., the higher the temperature, the faster the decarboxylation [65].

## 6. Potential Applications of Cannabis in the Food Industry

The possible cannabis applications in the food industry are very diverse and full of potential in the upcoming years. With the growing awareness of the natural and health benefits of hemp, cannabis use in the food industry is expected to expand. Some of the potential applications include:

### 6.1. New Foods with CBD

New food products enriched with CBD may appear in the future. Including CBD in foods may offer consumers additional ways to obtain the health benefits of this cannabinoid. New foods containing CBD may be diverse and interesting to consumers. CBD can be incorporated into a wide range of food products. For example, hemp energy bars could be a popular way to obtain a quick energy boost while enjoying the potential health benefits of CBD. Moreover, there is hemp muesli enriched with hemp seeds and CBD, and the already well-known, but not widely available, CBD-infused hemp chocolates. In addition to the products mentioned above, the use of CBD as a raw material in the production of hemp drinks or baked goods suitable for people suffering from celiac disease could also be considered [102].

### 6.2. Hemp Protein and Food for Vegans

Hemp is a rich source of protein and essential fatty acids, making it an attractive alternative to traditional proteins for vegans and vegetarians. In the future, new hemp foods specifically for these groups of consumers could emerge. Thanks to the content of hemp, people with a vegan diet can be provided with foods with a diverse and rich nutritional composition.

Particularly, hemp seeds are an important source of proteins, fiber, and essential fatty acids such as omega-3 and omega-6 fatty acids. They can be eaten raw, added to salads, muesli, and smoothies, or included in baked goods. Their other potential use is to be considered as a raw material for protein isolation. One of the other foods with potentially greater use is hemp milk as an alternative to conventional milk drinks [103].

### 6.3. Innovations in Hemp-Processing Technologies

The future may bring innovations in hemp-processing technologies that will allow the creation of new and improved hemp foods with better texture, taste, and nutritional value. Continuous advancement and development of hemp-processing technology are key factors in optimizing the production of high-quality, efficient, and sustainable hemp products. The rapidly growing interest in cannabinoids and other valuable components of cannabis is driving the need for new approaches and techniques that include [102]:

(a) Nanotechnology: This technology allows the particle size of cannabinoids and other valuable substances to be reduced to the nanometer level. It can increase their bioavailability and improve absorption efficiency, which has the potential to increase the potency of cannabis products.
(b) Supercritical CO_2_ extraction: It is a modern technique that allows the efficient and gentle extraction of cannabinoids, terpenes, and other valuable components from cannabis. This method allows the separation of cannabinoids with high purity and minimizes the use of solvents, making it a greener and safer alternative to traditional extraction methods.
(c) Fermentation: It may be a new way to extract valuable substances from cannabis, such as cannabinoids and enzymes. This method is more environmentally friendly and can reduce processing costs.
(d) Biotechnological methods: These methods can be used to optimize the production of specific cannabinoids and terpenes. Genetic modification of cannabis can lead to the development of varieties with higher levels of specific compounds and better resistance to pests.

Innovation in hemp-processing technology is an important factor in the development and diversification of the market for hemp products. With continued research and innovation, further improvements in processing technologies can be expected, leading to ever better and more efficient hemp products for various industries [102].

### 6.4. Functional Foods with Hemp

Functional foods with hemp are food products that offer not only nutritional value but also natural benefits for human health, due to the content of cannabinoids, terpenes, essential fatty acids, and other phytonutrients that contribute to the hemp’s functional properties. In recent years, interest in functional foods has increased and manufacturers are beginning to incorporate cannabis into a variety of functional food products to provide additional health benefits to consumers. Cannabinoids, in particular cannabidiol (CBD), are key components of cannabis with various pharmacological effects. Studies suggest that CBD has anti-inflammatory, antioxidant, anxiolytic, and neuroprotective properties. Incorporating CBD into functional foods may provide potential benefits for stress reduction and improved sleep and overall well-being [25,26].

Another possible use of cannabis in the production of functional foods is in combination with adaptogens. Adaptogens are herbal substances that help the body adapt better to stress and increase resilience. Combining cannabis with adaptogens such as ashwagandha or rhodiola can offer synergistic effects to reduce anxiety, increase resilience, and promote physical and mental well-being [104].

Finally, thanks to the nutrient-rich content of cannabis, functional foods containing it can be an important source of essential nutrients that are important for maintaining a healthy metabolism and overall well-being. Due to the combination of the high nutritional value of hemp and its phytonutrients, these foods can contribute to an overall balanced diet [105].

## 7. Future Directions of Cannabis Applications

Research on hemp’s application in the food industry should focus on several key factors to maximize the potential of the cannabis plant and ensure its safe and effective utilization in food products. These factors include:

1. Safety and regulation: Extensive studies are needed on the safety of cannabis and its constituents, such as cannabinoids and terpenes, when used in food. These studies should assess possible adverse effects, drug interactions, and long-term effects on human health [106].
2. Optimization of processing technologies: Research should focus on finding innovative and efficient processing technologies for the food industry. This may include improving extraction methods, determining optimal temperatures and times for cooking and baking with cannabis, and exploring different ways to incorporate cannabis into foods with minimal loss of its active ingredients [107].
3. Study of bioactive substances: Investigation should aim at the bioactive substances contained in cannabis and their potential effects on human health. This comprises studying the cannabinoids, terpenes, flavonoids, and other phytochemicals in cannabis and their effects on the human body, including their interactions with the endocannabinoid system [34].
4. New product development: Research should focus on the development of new hemp-based food products that offer interesting and attractive options for consumers. This involves the creation of new cannabis dishes, desserts, drinks, and other products that are both appealing and healthy [108].
5. Sustainability and environmental friendliness: The objective of research should be to find sustainable ways of growing and processing hemp for the food industry in order to minimize negative environmental impacts. This includes exploring options for organic cultivation, reducing water and energy consumption, and waste treatment [91].

## 8. Conclusions

In conclusion, the escalating research and interest in the utilization of CBD and hemp within the food industry are propelled by an expanding awareness of the potential health advantages inherent in these botanical entities. Cannabidiol (CBD) and diverse cannabinoids inherent in hemp manifest a diverse array of pharmacological effects, encompassing anti-inflammatory, analgesic, anxiolytic, and neuroprotective properties, thereby amplifying their prospective utility in augmenting human health. In this review, it was revealed that in the food industry hemp appears to be an attractive source of innovation and opportunity. Incorporating hemp elements into food products, from hemp oils, proteins, and fibers to hemp tea and hemp milk, can bring significant benefits. CBD, a key ingredient in selected products, serves as an alternative to conventional ingredients and has the potential to enhance human health and well-being. This combination of scientific research and growing applications underscores the promising trajectory of CBD and hemp in today’s culinary environment and heralds a paradigm shift in nutrition and pharmacology.

## Data Availability

Data sharing is not applicable to this article.
